# Attachment and Substance Use Disorders—Theoretical Models, Empirical Evidence, and Implications for Treatment

**DOI:** 10.3389/fpsyt.2019.00727

**Published:** 2019-10-15

**Authors:** Andreas Schindler

**Affiliations:** Department for Personality and Stress Disorders, Center for Psychosocial Medicine, University Medical Center Hamburg-Eppendorf, Hamburg, Germany

**Keywords:** Attachment, attachment theory, patterns of attachment, substance use disorders, substance abuse, addiction

## Abstract

**Introduction:** The article reviews attachment-oriented research in individuals with substance use disorders (SUDs). Based on attachment theory, substance abuse can be understood as “self-medication,” as an attempt to compensate for lacking attachment strategies. Attachment theory suggests a developmental pathway from insecure attachment to SUD and, on the other hand, a negative impact of substance abuse on attachment security. Earlier reviews have indicated a general link but have been inconclusive with regard to other aspects. In the light of a growing body of research, this review is looking for evidence for the general link, for its direction, for differences due to different patterns of attachment, different substances and severities, comorbid psychiatric disorders, and age groups.

**Methods:** Using medical and psychological databases, 34 cross-sectional studies, three longitudinal studies, and a systematic meta-analysis were identified. Methodological problems such as poor assessment of SUD and the use of different measures of attachment limit comparability.

**Results:** All cross-sectional studies in the review confirm a link between insecure attachment and SUD. Results of longitudinal studies show insecure attachment to be a risk factor for SUD, while continued substance abuse impairs the ability to form close relationships. With regard to specific patterns of attachment, results mainly point toward very insecure patterns. They indicate different patterns of attachment in different groups of substance abusers, suggesting different developmental pathways. Fearful–avoidant attachment was frequent in heroin addicts, while alcohol abusers displayed more heterogeneous patterns. Comorbid mental disorders and severity of SUD seem to be important factors, but data are still inconclusive. The link between insecure attachment and SUD seems to be stronger in adolescence compared to adulthood.

**Discussion:** The last decades have seen a substantial growth in studies on attachment and SUDs. Despite methodological problems, the general link between insecure attachment and SUD today is well established. Attachment theory might contribute to the understanding and treatment of SUDs in a significant way. But to do so, a lot of open questions have to be answered. We will need more carefully designed longitudinal studies, more studies connecting psychological data with brain processes, and more clinical trials.

## Introduction

Over the last decades, attachment theory (see **BOX 1** for a brief description of attachment theory) has been applied to a lot of developmental and clinical fields, including substance use disorders (SUDs). A growing number of attachment-based studies have tried to contribute to the understanding of SUDs. In 2005, a first review tried to structure the field (1). It contained two main questions:

1. Is there a link between attachment and SUD?
2. Is there a link between one or several specific attachment patterns and SUD?

Additionally, it asked for the direction of these possible links, that is, for developmental pathways between attachment and SUD. It looked for differences between different age groups, between users of different substances, due to different levels of severity of SUD (use, abuse, addiction) and due to different comorbid psychiatric disorders. This first review identified 12 studies published between1990 and 2005. Results indicated a link between insecure attachment and SUD, but they were inconclusive with regard to any other question. In the light of a growing body of research, this article is going to readdress the questions of the 2005 review. It tries to give a concise overview over what we know today about individual patterns of attachment among consumers of psychotropic substances. This might help to prepare the ground for a possible later integration of attachment in a multifactorial model of SUDs [see West and Brown (2), for an overview over addiction theories] and in the treatment of SUDs. Note that this review will not cover the topic of addictive behaviors such as gambling disorder or internet gaming disorder. And it will not cover the vast body of research on attachment in children of substance-abusing parents. This article will first give a theoretical introduction and sum up what we know from earlier reviews. It will then move on to methodological issues and to a review of the evidence represented in empirical studies today.

### Insecure Attachment and Substance Use Disorders

Human beings who do not experience a sufficiently secure base develop insecure patterns of attachment, including negative IWMs of themselves and others, and negative expectations with regard to relationships (this includes therapeutic relationships, making it more difficult to establish a treatment alliance). Although insecure attachment is not a pathological condition in itself, it is related to mental disorders. Its ratio in clinical samples is 86%, in contrast to 42% in the general population (9). It is seen as an important risk factor not only for SUD, but also for mental disorders in general (10). With increasing insecurity, individuals will face more difficulties in regulating emotions and stress. This regulation will not function either with the help of attachment figures or with the use of IWMs. At the same time, insecure individuals will face difficulties in forming and maintaining relationships with others. Psychotropic substances then might become attractive as one way to “self-medicate” attachment needs, to regulate emotions, to cope with stress, and to replace relationships (8, 11, 12). Earlier reviews have shown cross-sectional evidence for a link between insecure attachment and SUDs (1, 8, 10, 13, 14). Additionally, they have reported preliminary longitudinal evidence for insecure attachment being a risk factor for later substance abuse. This review will look for a replication of the general link and for more longitudinal data.

Box 1 **What is attachment?**

“Attachment is a motivational, behavioral, and interactional system that provides security for immature offspring in a variety of species. The attachment system regulates distance and closeness of parents (or ‘attachment figures’) and children. The child will seek closeness to his/her parents whenever he/she feels in danger. Ideally, parents will then comfort the child, calm him/her down, and give him/her a rewarding feeling of security. This feeling of security or ‘secure base’ created in early attachment experiences helps the child to regulate his/her emotions and is an important step on the way to acquiring own coping strategies when facing fear or distress. Against the backdrop of a ‘secure base’, the child can explore his/ her environment (3–5). At the same time, secure attachment is the base for an exploration of his/her own inner world and that of others, that is, for the ability to ‘mentalize’ and to gain a coherent picture of mental processes (6). Over time, experiences with attachment figures are internalized. The child develops cognitive representations [‘inner working models’ (‘IWMs’)] of himself/herself and of his/her attachment figures. If positive IWMs are developed, other persons than the original attachment figures can also become a secure base. Additionally, positive IWMs make it possible to regulate affective states autonomously without depending on another person. In this sense, ‘secure attachment liberates’ (7).” (8, p. 305).

Although most theoretical and empirical work has focused on insecure attachment as a risk factor for the development of SUDs, it is likely that substance abuse has an effect on attachment, too. The consequences of substance abuse are a host of well-known developmental risks and neurological impairments (15). From an attachment perspective, four mental processes might be directly affected by substance abuse. First, exploration of the environment is reduced or distorted, or risks are taken that would never have been taken in a state of sobriety (16). Second, mentalization, the exploration of the inner, mental world of oneself and others is reduced (17). This might even be a possible motivation for substance abuse: nonmentalization and nonperception of distress and painful memories. Third, age-appropriate experiences in relationships often are inhibited or even prevented (18). Fourth, affect regulation and reward might be replaced by substance abuse (19). Further indirect evidence comes from the host of studies in samples of substance-abusing parents. These parents are hardly able to establish secure attachment relationships with their offspring (20). In sum, substance abuse might well have a negative impact on the ability to attach and form close relationships. Earlier reviews failed to provide empirical evidence regarding this point. This review will look for longitudinal evidence for an impact of substance abuse on attachment.

### Individual Patterns of Attachment and SUDs

**Figure 1:**
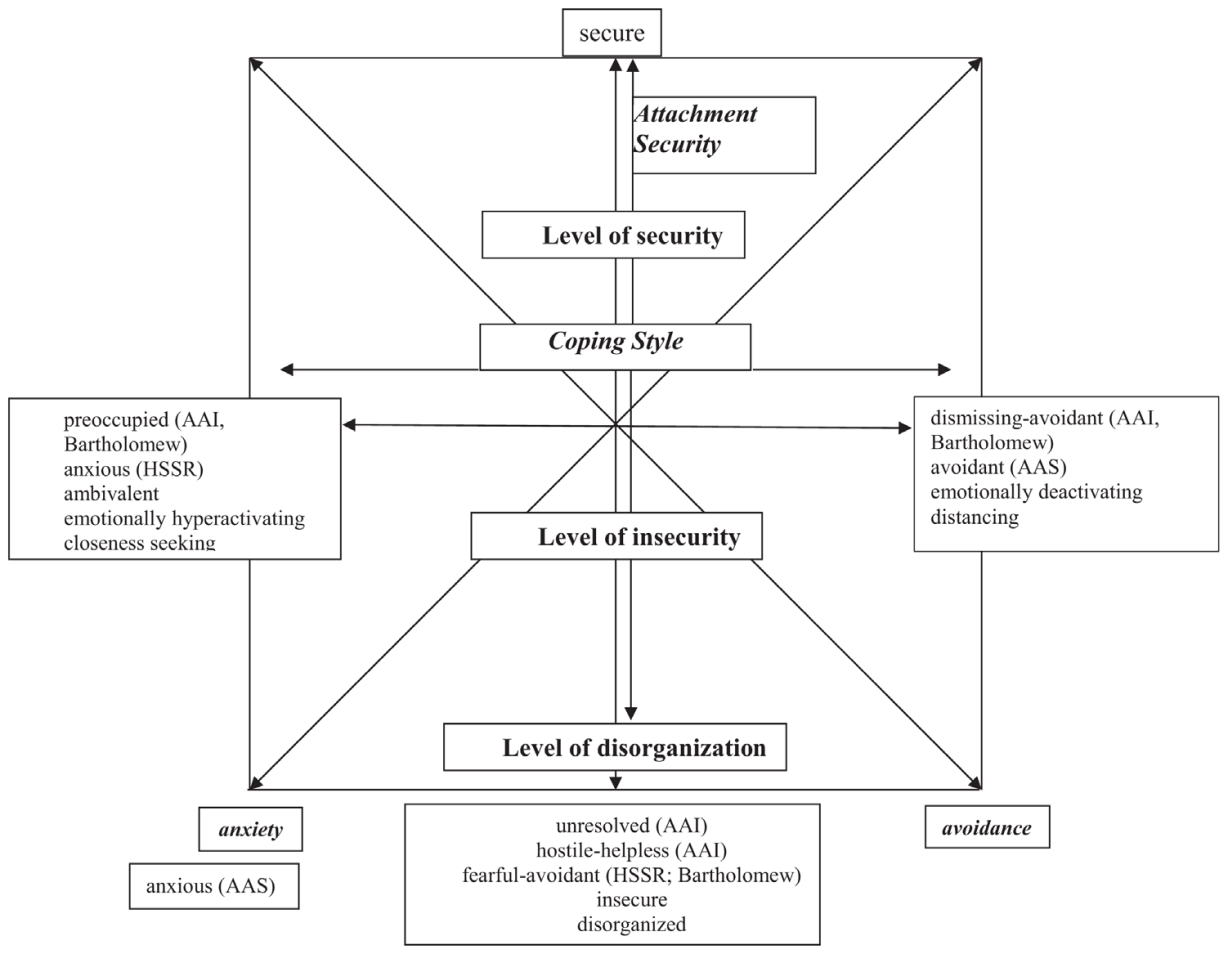
Two-dimensional model of attachment.

Attachment theory describes different patterns, which are based on the specific experiences in attachment relationships. They involve different levels of security, different strategies of coping with negative experiences in close relationships, and different means of regulating negative affect and expressing attachment needs. Individuals with preoccupied (sometimes called ambivalent/enmeshed/anxious) patterns use affectively hyperactivating strategies and are seeking closeness to important others. They are preoccupied with their own distress and the availability of attachment figures. Individuals with dismissing–avoidant strategies, on the other hand, tend to use distancing, affectively deactivating strategies. They defensively turn their attention away from their emotional distress and their attachment figures. A third group of patterns is characterized by a lack of functioning coping strategies and the highest risk for the development of severe psychopathology: disorganized patterns of attachment. These are associated with parental psychopathology (SUDs among others), with traumatic experiences (sexual abuse and maltreatment) as well as loss and neglect (21). While attachment originally described these patterns as categories, a dimensional approach seems to represent the existing data more precisely (22). **Figure 1** presents a two-dimensional model of attachment patterns, trying to integrate the different constructs. Note that this model is only meant to give a rough orientation. The dimension secure–insecure is well established. Especially the definition of secure attachment is common ground. However, there are very different concepts describing the insecure end of this dimension (disorganized, unresolved, fearful–avoidant, hostile–helpless). Although these concepts are different, they share the lack of adaptive coping strategies and a high risk for developing mental disorders. The second dimension is generally labeled “coping style” with preoccupied patterns on the left-hand side and dismissing–avoidant patterns on the right-hand side. Two-dimensional models of attachment patterns often use the dimensions of anxiety and avoidance (23). This is a factor solution that is rotated by 45° to the one described here (**Figure 1**). For more detailed discussions of these concepts, see Ravitz et al. (24) and Shaver and Mikulincer (22).

Evidence presented in earlier reviews was inconclusive with regard to specific patterns of attachment. While some studies pointed to more avoidant patterns in substance abusers (1), others indicated links with different patterns (8, 10, 14). There had not been any longitudinal data on possible developmental pathways from specific patterns toward SUD. The relation between specific patterns and SUD is still an open question to be addressed in this review.

### The Use of Different Substances

According to the “self-medication hypothesis” (12), the abuse of specific substances might be an attempt to cope with specific forms of emotional distress. For example, the abuse of stimulants might be linked to more hyperactivating, closeness-seeking attachment strategies, while the use of sedatives might be linked to deactivating, distancing strategies. Following the opioid deficit hypothesis (25; see **Box 2**), abuse of heroin and other opioids might be linked to extremely insecure attachment.

Box 2 **Neurobiological research and the reward–deficiency hypothesis.**

Neurobiological research has focused on motivational processes of both attachment and substance abuse (26–29). Both are transmitted by the same mesolimbic and mesocortical circuits, and for both, dopamine, endorphins, oxytocin, and vasopressin play important roles. This line of research mainly relies on the reward–deficiency hypothesis of addiction (30, 31), assuming that psychotropic substances can substitute other “deficient” sources of reward. Attachment theory posits that insecure individuals have not sufficiently experienced the reward of a secure base. Their reward system tends to be insufficiently conditioned to satisfaction by social contact (29). Based on a host of animal studies on endorphins and opioids, Trigo et al. (25) have operationalized reward–deficiency as an opioid deficit. They assume that insecure attachment and insufficient conditioning to reward by social contact lead to a lack of endorphins in the VTA. As a consequence, dopaminergic reward processing in the limbic system cannot be released. This leads to a reward deficiency and increases the risk for addictive behaviors. Especially opioids might be a potent substitute for lacking attachment strategies. Recently, Alvarez-Monjaras et al. (19) have presented a multifactorial developmental model of attachment and addiction. The model basically assumes a functional interchangeability of attachment processes and substance use. According to this model, positive attachment experiences and secure patterns strengthen reward from social contact and decrease the risk for addictive behaviors. Negative attachment experiences and insecurity, on the other hand, lead to insufficient reward from social contact and to a heightened risk to replace it with addictive behavior (19).

Despite some data from studies in alcohol and heroin using samples, earlier reviews have been inconclusive. The question of attachment-related differences between users of different substances will have to be addressed in this article.

### Severity of Substance Use

In theory, more insecure individuals face a higher risk for developing SUDs. This does not necessarily imply that they develop more severe forms of SUDs. But if substance abuse impaired the attachment system, severity of abuse might be linked to severity of impairment. The review by Iglesias et al. (14) reported some evidence for a difference between experimental substance *use* and substance *abuse* in adolescent samples. The evidence in earlier reviews is limited, so it is still an open question: Does severity of substances use (use, abuse, addiction) make a difference with regard to attachment?

### Comorbid Psychiatric Disorders

Comorbid psychiatric disorders are common in samples of substance abusers. Insecure attachment is not exclusively related to SUDs but to psychiatric disorders in general (10). Comorbidity might well be an important mediator of findings in this area. At the same time, it makes research very complex, because individuals with different comorbid disorders might use different substances for different reasons.

Schindler et al. (1) presented some limited evidence for different patterns of attachment in substance-abusing adolescents with different comorbid disorders. However, the question of the role of comorbid disorders in the relation between attachment and SUDs has to be readdressed.

### Age: Substance Abuse in Adolescence Vs. Adulthood

The use and abuse of psychotropic substances usually begin and peak in adolescence. It is a crucial phase for the development of SUDs (11). At the same time, adolescence is important in the development of attachment. It is a transitional period when autonomy from parents, from the “secure family base,” is developed (32, 33). This might suggest a closer relation between attachment and SUD in adolescence than in adulthood. Two earlier reviews have discussed these complex topics in detail (8, 14) but have not presented any data comparing adolescent and adult samples. This review will look for age-related effects with regard to attachment and SUDs.

## Methods

**Figure 2:**
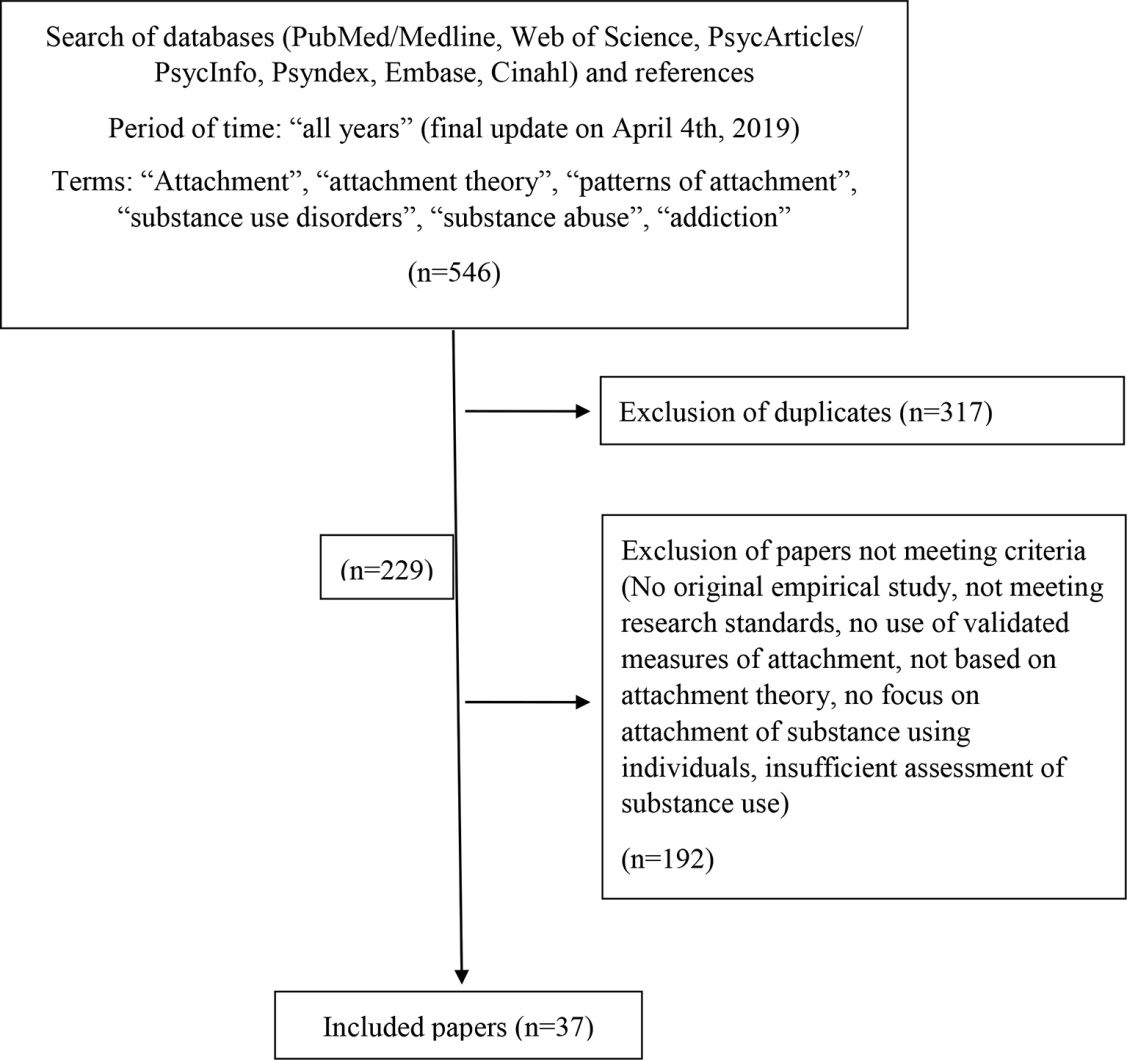
Flowchart study selection.

Literature for this review was scanned in PubMed/MEDLINE, Web of Science, PsycARTICLES/PsycINFO, PSYNDEX, EMBASE, and CINAHL databases for “all years” with a final update on April 4, 2019, using the following keywords: “attachment,” “attachment theory,” “patterns of attachment,” “substance use disorders,” “substance abuse,” and “addiction.” Additionally, references in articles and presentations were tracked. Criteria for inclusion were original empirical studies; basic research standards are met (which was not the case in studies earlier than 1990); use of validated measures of attachment; study based on attachment theory; focus on attachment of substance using individuals (this excluded studies focusing on children of substance users); and assessment of substance use, abuse, or addiction. Five hundred forty-six publications were scanned. After removing duplicates and studies not meeting the criteria, we included 37 original studies on attachment and SUD and one quantitative meta-analysis. Three of the original studies were longitudinal. Two further studies had a longitudinal design, but reported only cross-sectional data for the question at hand. See **Figure 2** for a flowchart of the selection process.

Although we only included studies grounded in attachment theory, the use of different attachment measures makes results difficult to compare results. Additional methodological problems arise from flaws in the assessment of substance abuse and in sample selection. Samples were very heterogeneous, including different substances and different stages of severity. Most studies relied on self-report measures of substance use, with urinalyses or similar physical measures being rare.

### Measures of Attachment Patterns

Attachment research has developed different measures. These share the basic distinction between secure and insecure attachment, but differ in the definition and labeling of specific patterns. While attachment interviews assess attachment representations, defined as the state of mind with regard to early attachment experiences, self-report questionnaires assess attachment styles, defined as experiences and behavior in close relationships (including romantic relationships). Although attachment theory assumes that these patterns develop in early childhood, both types of measures assess the current state of the attachment system. Attachment questionnaires and interviews show moderate correlations. The majority of studies use self-reports, which are seen as “surface indicators” of attachment representations (22, 24). The Adult Attachment Interview (AAI) (34) is a semistructured interview with four categories: secure–autonomous, preoccupied, dismissing, and unresolved. The category “hostile–helpless” was added later to describe special patterns mainly occurring in clinical samples (35). The Adult Attachment Projective (AAP) (36) is a projective test designed to produce narratives that can be categorized in the same way as the AAI. The Hazan and Shaver Self-report (HSSR) (37) is a simple measure consisting of brief descriptions of three attachment styles with respect to experiences in romantic relationships. Attachment styles are called secure, anxious–ambivalent, and avoidant. Note that avoidance is rather defined as fearful–avoidance in the Bartholomew model (high insecurity, no coping) and not as dismissing avoidance in the AAI. The Adult Attachment Scales (AAS) (38) is a multi-item scale based on the HSSR. It assesses secure, anxious, and avoidant attachment styles. Note that anxious attachment here is defined as the high end of the anxiety scale. Bartholomew and Horowitz (23) developed a model of four attachment categories, based on positive and negative internal working models of the self and of others. Bartholomew differentiated between two avoidant categories: fearful–avoidant (according to the HSSR) and dismissing–avoidant (according to the AAI) (**Figure 1**). Based on this model, several self-report measures such as the Relationship Questionnaire, the Relationship Scale Questionnaire (39), the Experiences in Close Relationships (40), and an Attachment Interview have been developed (23).

## Results

### Insecure Attachment and SUDs

All studies in this review report a link between insecure attachment and substance abuse or addiction (**Table 1**). Secure attachment was typically found in healthy controls in all studies including a control group. Cooper et al. (41) additionally showed a relation with experimental substance use in adolescence.

**Table 1:** Studies on attachment and substance use disorders.

Authors, Year	Age group	Sample	N/controls	Substances	Severity	Method	Measure of attachment	Main attachment pattern
Delvecchio et al. (42)	Adult	Clinical, TSUD	40/—	Illicit drugs	Addiction	CS	AAP	Unresolved
Amman (43)	Adolescent	Clinical, TSUD	15/15	Unspec.	Addiction	CS	AAI	Dismissing, cannot classify, unresolved
Branstetter et al. (44)	Adolescent	Nonclinical	200/—	Unspec.	Abuse	Long.	AAI, HSSR	Insecure (mediated by maternal monitoring)
Melnick et al. (35), Finger (45)	Adult	Clinical, TSUD	62/87	Heroin	Addiction	CS	AAI	Hostile–helpless
Caspers et al. (46), Caspers et al. (47)	Adult	Nonclinical, adoptees	208/—	Unspec.	Use/abuse	CS	AAI	Insecure
Riggs and Jacobvitz (48)	Adult	Nonclinical, expect. parents	233/26	Unspec.	Abuse	CS	AAI	Unresolved
Fonaghy et al. (49)	Adult	Clinical, psychiatric	82/37	Unspec.	Abuse	CS	AAI	Unresolved, preoccupied
Rosenstein and Horowitz (50)	Adolescent	Clinical, psychiatric	60/29	Unspec.	Abuse	CS	AAI	Dismissing, preoccupied
Allen et al. (51)	Adolescent	Clinical, psychiatric	66/76	Illicit dr.	Abuse	CS (Long.)	AAI	Dismissing
Finzi-Dottan et al. (52)	Adult	Clinical, TSUD	56/56	Heroin	Addiction	CS	HSSR	Avoidant
Cooper et al. (41)	Adolescent	Nonclinical	2011/1151	Unspec.	Use/abuse	CS	HSSR	Secure vs. anxious
Mickelson et al. (53)	15–54 y	Nonclinical, representative	8089/2876	Unspec.	Abuse	CS	HSSR	Avoidant (anxious)
Brennan and Shaver (54)	Young adult	Nonclinical, college	242/178	Alcohol	Use	CS	HSSR	Avoidant
Senchak and Leonard (55)	Young adult	Nonclinical	644/—	Alcohol	Use/abuse	CS	HSSR	Men: avoidant, women: unrelated
Durjava (56)	Adult	Clinical, TSUD	54/54	Heroin	Addiction	CS	AAS	Insecure
Unterrainer et al. (57)	Adult	Clinical, TSUD	19/40	Heroin	Addiction	CS	AAS	Anxious
Mortazavi et al. (58)	Adult	Clinical, TSUD	60/60	Opium	Addiction	CS	AAS	Insecure
Shin et al. (59)	Adult	Nonclinical, male	141/—	Alcohol	Abuse	CS	AAS	Anxious
Kassel et al. (60)	Young adult	Nonclinical, college	212/—	Unspec.	Abuse	CS	AAS	Anxious
Vaz-Serra et al. (61)	Adult	Clinical, TSUD, male	56/56	Alcohol	Addiction	CS	AAS	Anxious
Gidhagen et al. (62)	Adult	Clinical, TSUD	108/—	Unspec.	Addiction	CS	BSR	Fearful (preoccupied, dismissing)
Le et al. (63)	Adult	Primary care	348/—	Alcohol	Abuse	CS	BSR	Anxiety dimension
Schindler and Sack (64)	Adult	Clinical, psychiatric	36/21	Unspec.	Abuse/Addiction	CS	BAI	Fearful (dismissing)
Wedekind et al. (65)	Adult	Clinical, TSUD	59/—	Alcohol	Addiction	CS	BSR	Insecure
Jenkins and Tonigan (66)	Adult	Alcoholics Anonymous	253/—	Alcohol	Addiction	CS (Long.)	BSR	Anxiety dimension
Harnic et al. (67)	Adult	Clinical, TSUD	40/—	Alcohol	Addiction	CS	BSR	Preoccupied
Molnar et al. (68)	Young adult	Clinical, TSUD	213/696	Alcohol	Abuse	CS	BSR	Fearful (preoccupied, dismissing)
DeRick and Vanhuele (69), DeRick et al. (70)	Adult	Clinical, TSUD	101/—	Alcohol	Addiction	CS	BSR	Insecure
Schindler et al. (71)	14–29 y	Clinical, TSUD	94/72	Heroin/XTC/THC	Addiction/abuse	CS	BAI	Fearful vs. insecure vs. dismissing
Doumas et al. (72)	Adult	Clinical, TSUD	46/—	Unspec.	Addiction	CS	BSR	Fearful (preoccupied, dismissing)
Thorberg and Livers (18)	Adult	Clinical, TSUD	99/58	Unspec.	Addiction	CS	BSR	Fearful (preoccupied, dismissing)
Schindler et al. (1)	14–25 y	Clinical, TSUD	71/71	Heroin	Addiction	CS	BAI	Fearful
Vungkhanching et al. (73)	Young adult	Nonclinical, College	369/—	Alcohol	Abuse	CS	BSR	Fearful (preoccupied, dismissing)
McNally et al. (74)	Young adult	Nonclinical, College	366/366	Alcohol	Use	CS	BSR	Fearful (preoccupied)
Zeid et al. (75)	Adult	Clinical, TSUD	149/92	Alcohol/. opiates	Addiction	CS	ACIQ	Insecure (no difference between groups)
Zhai et al. (76)	10–22 y	Nonclinical	694/—	Unspec.	Abuse	Long.	YAPS	Insecure
Danielsson et al. (77)	Adolescent	Nonclinical, community	1222/—	Unspec.	Use/abuse	Long.	IPPA	Insecure

TSUD, treatment of SUD; Unspec., substances not specified; CS, cross-sectional; Long., longitudinal; XTC, ecstasy; THC, cannabis; AAI, Adult Attachment Interview; AAP, Adult Attachment Projective; AAS, Adult Attachment Scale; ACIQ, Attachment and Clinical Issues Questionnaire; BSR, Bartholomew Self-report (RQ, RSQ, ECR); BAI, Bartholomew Attachment Interview; HSSR, Hazan & Shaver Self-report; IPPA, Inventory of Parent and Peer Attachment; YAPS, Youth Attachment to Parents Scale.

Three longitudinal studies indicate that attachment in an earlier age has an impact on later substance abuse. Branstetter et al. (44) demonstrated that securely attached adolescents at age 14 years consumed fewer substances at age 16 years. Danielsson et al. (77) showed that attachment security at age 13 years prevented heavy drinking episodes at age 15 years. In a study by Zhai et al. (76), insecure attachment at age 10 to 12 years led to dysregulation at age 16 years and substance abuse at age 22 years. In a meta-analytic calculation, Jordan and Sack (78) calculated that secure attachment decreases the risk for substance abuse by odds ratios ranging from 0.60 to 0.70. Thus, the risk for substance abuse is about one-third lower for securely attached adolescents.

The impact of substance abuse on attachment security has been studied less frequently. Unterrainer et al. (57) found such an impact with a strong neurotoxic effect in a clinical study of long-term addicts. Nonclinical studies have been less conclusive (79). A recent quantitative meta-analysis of prospective longitudinal studies (80) analyzed 34 original studies with as many as 56,721 participants. Studies mainly investigated community or college samples with a mean age of 15 years (range, 7–30 years); they covered a mean period of time of 3.8 years, and they mainly used attachment self-reports. The analysis yielded significant prospective relations in both directions with a significantly stronger effect of insecure attachment on substance abuse than *vice versa*.

### Individual Patterns of Attachment: Styles and Representations

Data from longitudinal studies do not provide any information about different developmental pathways of individuals with specific patterns of attachment. However, the last three decades have seen a substantial growth of cross-sectional studies. Eight studies were carried out with the AAI/AAP. Six used the HSSR, and another six the AAS. Fourteen studies used measures based on the Bartholomew model. Three studies used other measures Attachment and Clinical Issues Questionnaire, Youth Attachment to Parents Scale, Inventory of Parent and Peer Attachment (ACIQ, YAPS, IPPA). Before describing results in detail, here is a brief overview:

- AAI/AAP studies mainly show dismissing and unresolved representations.
- In HSSR studies, fearful attachment was the most frequent style.
- AAS studies mainly report anxious attachment styles.

The majority of studies used the Bartholomew model point toward fearful–avoidance, with some evidence for a link with the anxiety dimension.

#### AAI/AAP Studies

A small German study (43) found dismissing and unresolved representations in adolescent drug addicts using multiple substances. Two other studies examined samples of adolescents in psychiatric inpatient treatment with SUD and other psychiatric diagnoses. Rosenstein and Horowitz (50) found partly dismissing and partly preoccupied representations in substance abusers with different comorbid disorders. Allen et al. (51) report a relation between “hard drug use” and dismissing attachment. Although this study had a longitudinal design, results concerning attachment and SUD were cross-sectional. Studies in adult samples found hostile–helpless representations (35, 45) among African American mothers in methadone maintenance treatment, a general link to insecurity in a sample of adults who had been adopted in childhood (46, 47) and unresolved representations among expecting parents (48), among substance-abusing psychiatric inpatients (49), and among adult drug addicts (using the AAP; 42).

#### HSSR Studies

HSSR studies mainly examined nonclinical samples. While a high-school study reported a link between anxious attachment and “problematic” substance abuse (41), the majority of substance abusers in a large representative US-wide sample described themselves as avoidant (53). So did the majority of “heavy drinkers” in college (54) and young adult samples (55), as well as adult long-term heroin addicts in Israel (52).

#### AAS Studies

Most AAS studies report anxious attachment in substance-abusing college students (60), in alcohol abusers in Korea (59), in alcohol addicts (61), and heroin addicts (57). An exception is the study by Durjava (56), which reports heightened scores on all insecure scales in heroin addicts.

#### Studies Using Measures Based on the Bartholomew Model

Studies in college samples mainly found links between alcohol abuse and fearful–avoidant patterns, while preoccupied and dismissing patterns occurred less frequently (68, 73, 74). The same constellation of patterns were found in clinical samples of substance-dependent individuals (18, 62, 64, 72). In samples of heroin addicts, fearful–avoidant attachment was the main pattern (1, 52, 71), while alcohol addicts showed either preoccupied (67) or generally insecure attachment (65, 69, 70). A study in adults in primary care found hazardous drinking to be linked to the anxiety dimension (63). Jenkins and Tonigan (66) found elevated attachment anxiety in an Alcoholics Anonymous (AA) sample. Although this study had a longitudinal design, results concerning attachment, and SUD were cross-sectional.

### Different Substances

Only two studies compare users of different substances systematically. Zeid et al. (75) did not find any differences between alcohol and opiate addicts. In contrast to this study, Schindler et al. (71) did find significant differences between heroin, ecstasy, and cannabis users and nonclinical controls. While heroin addicts were mainly fearful–avoidant, controls were mainly secure, and cannabis abusers tended to be dismissing–avoidant. Ecstasy (MDMA) abuse was related to insecure attachment, but not to a specific attachment pattern.

Studies in specific groups provide some additional information about heroin, alcohol, and cigarette smoking. With regard to heroin addiction, they indicate fearful–avoidance (1, 52), as well as hostile–helpless representations in the AAI (45) and insecurity in general in the AAS (56). Studies in samples of alcohol users also showed avoidant and highly insecure patterns, but higher rates of preoccupied/ambivalent attachment (67) and a relation with the anxiety dimension, too (59, 61, 63, 66). The meta-analysis of Fairbairn et al. (80) shows a close relation between attachment-based emotion regulation and cigarette smoking.

### Severity of Substance Use

A comparison of studies in clinical versus nonclinical samples does not show any systematic differences in attachment patterns. Especially alcohol use, abuse, and addiction have been studied repeatedly without finding different patterns of attachment. However, results show a correlation between severity of opioid addiction and attachment insecurity. Opiate addicts in Iran were more insecure than nonaddicted opiate users (58). Severity of heroin use correlated with fearful–avoidant attachment (1, 62).

### Comorbid Psychiatric Disorders

Rosenstein and Horowitz (50) report mainly dismissing classifications in adolescent substance abusers with comorbid conduct disorders but partly dismissing and partly preoccupied classifications in those with affective disorders. In a study of Schindler and Sack (64), comorbid patients with SUD and borderline personality disorder (BPD) were similar to other BPD patients in several psychiatric measures, but closer to SUD patients with regard to attachment. They were more avoidant and less preoccupied than other BPD patients. With regard to PTBS, three studies found a link between SUDs and unresolved attachment (43, 48, 49), while two other studies did not find this relation in adolescent samples (50, 51).

### Age: Adolescent vs. Adult Samples

The meta-analysis by Fairbairn et al. (80) shows a closer relation between insecure attachment and substance abuse in adolescents than in adults. In AAI studies in adolescent samples, dismissing attachment seems to be the most frequent representation, while adult samples mainly showed unresolved and hostile–helpless representations. Other studies do not indicate any systematic differences between adolescent and adult samples.

## Discussion: Implications for Research and Treatment

### Insecure Attachment and SUDs

A host of cross-sectional studies consistently replicated the finding of a general link between insecure attachment and SUDs. Secure attachment is only occurring in experimental substance users and in healthy controls. Evidence from psychological studies is in tune with neurobiological findings. Longitudinal studies and meta-analyses indicate that secure attachment is a protective factor against substance abuse, and insecure attachment is a risk factor for substance abuse. Taken together, the general link between insecure attachment and SUDs today is well established, and there is moderate to strong evidence for the assumption of insecure attachment being a risk factor for SUD.

Additionally, there is moderate meta-analytic longitudinal evidence for a negative impact of substance abuse on attachment. This effect might be linked to the severity of substance abuse. The study by Unterrainer et al. (57) suggests that it might be, at least in part, an unspecific effect of neurotoxic impairments caused by substance abuse. The negative psychological effects described above might have an impact, too, but there is no direct evidence in the studies reviewed. Indirect evidence comes from parenting studies, showing that substance abusers have serious problems to provide secure attachment for their offspring (20). In the light of existing data, a vicious circle between insecure attachment and substance abuse seems likely. But we will need more longitudinal studies to gain a more detailed picture of this interaction. Studies will have to use psychological as well as neurobiological measures to control for possible confounds.

### Different Patterns of Attachment

It is more difficult to summarize the results of the 37 studies analyzing attachment patterns.

Their results mainly point toward very insecure patterns (unresolved–disorganized and hostile–helpless in the AAI, fearful–avoidant in the Bartholomew model). This supports the hypothesis of substance abuse as a substitute for deficient attachment strategies. But there is some evidence for other patterns as well, with avoidant patterns occurring more frequently than preoccupied or anxious ones. We still lack longitudinal data on developmental pathways from specific patterns toward SUD. Additionally, the selection of very different samples and the use of different measures make it difficult to draw conclusions. Differences between studies using different measures suggest a methodological bias. We need studies comparing different measures in one sample to discern these effects. Nonetheless, a lot of studies report different patterns within one sample, assessed with one measure. This suggests that different patterns are linked to SUD. From an attachment theory point of view, it seems likely that individuals with different patterns of attachment use psychotropic substances for different reasons. Individuals with preoccupied attachment might use substances to minimize social fears and to make it easier to get in touch with others. Individuals with avoidant patterns might use substances to avoid feeling negative emotions, attachment needs, and loneliness. Individuals with disorganized patterns might use substances to cope with fear and posttraumatic symptoms. Future research will have to consider different and complex pathways in a longitudinal design.

### Different Substances

Results from two systematic comparisons of users of different samples are inconclusive. There is some evidence for a link between heroin use and extremely insecure patterns. Although studies used different measures, all found these extremely insecure patterns, ranging on the level of disorganization (**Figure 1**). This is in tune with the endorphin-deficit hypothesis (25), assuming that opioids might be especially attractive for highly insecure individuals. Preliminary data on alcohol abuse point to different patterns. Studies found relations with avoidant and highly insecure as well as preoccupied/ambivalent patterns. It seems possible that alcohol abuse can have different functions. It might be used to reduce social fears and support closeness seeking in preoccupied individuals. Avoidant or fearful individuals, on the other hand, might use higher doses to avoid contact and deactivate emotions. The only study exploring ecstasy (MDMA) expected a relation with preoccupied attachment but found generally insecure patterns. The “entactogenous” effect of ecstasy does not seem to be related to closeness seeking in the sense of attachment. Meta-analytic data point toward a relation between nicotine and affect–regulation in adolescence. In mainly nonclinical samples, cigarettes might be the drug of choice for those with insecure attachment and problems to regulate emotions. Research on different substances is still fragmentary. Several important substances (e.g., cocaine, benzodiazepines, methamphetamines, etc.) have not even been studied. Systematic comparisons are rare. Although it is too early to report any definite relations, there does not seem to be a general link between substance abuse and a single specific pattern of attachment. This renders future research more complex, facing a variety of substances and patterns of consumption. We will need more systematic comparisons of different groups. Studies should include neurobiological data, considering different substance-related effects.

### Severity of Substance Abuse

Data on the severity of substance abuse are inconclusive, too. Whereas a comparison of samples of alcohol abusers versus addicts did not show any systematic differences, three studies report a correlation between severity of opioid addiction and attachment insecurity. This is in tune with theoretical models, and it might hint at the special role of opioids. However, we need more studies to draw conclusions.

### Comorbidity

Studies have addressed depressive, anxiety, conduct, borderline, and posttraumatic disorders, but we still lack knowledge from other important fields such as psychotic or bipolar disorders. Some studies showed different attachment patterns in substance abusers with comorbid conduct versus affective disorders. Another study reported differences between borderline patients with or without SUD. Posttraumatic stress disorders are special because they are linked to the concept of unresolved attachment and because clinical SUD samples show high rates of traumatic experiences (81). However, existing data on unresolved attachment and SUD are inconclusive. We still lack systematic studies on the relations between SUD, trauma, and unresolved attachment. Results on comorbid disorders in general show their relevance and the complexity of possible interrelations between attachment, SUDs, and comorbid disorders. But it is too early to draw any specific conclusions. Future research in clinical samples will generally have to take comorbidity into account.

### Age

Cross-sectional studies do not indicate any systematic differences in attachment patterns between adolescent and adult samples. The differences found in AAI studies are difficult to explain. However, meta-analytic findings of a closer relation between attachment and SUD in adolescence are more conclusive and more in tune with expectations. They underpin the importance of the developmental phase. Adolescence should be a focus of future research within a developmental framework. Because of the significance of the family background, this research will have to include a family systems perspective (**Table 2**).

**Table 2:** Review: questions, results, and implications.

Question	Results	Level of evidence	Research implications	Treatment implications
Is there a general link between insecure attachment and SUDs?	Yes	Strong (36 studies, meta-analytic data)	Explore underlying mechanisms	Consider insecure attachment in the treatment of SUD
Is insecure attachment a risk factor for SUDs?	Yes	Moderate–strong (three longitudinal studies, meta-analytic data)	Prospective longitudinal studies to explore developmental pathways	Attachment-oriented prevention for high-risk groups
Is substance abuse a risk factor for insecure attachment?	Yes, possible confound: neurotoxicity	Moderate (meta-analytic data, one additional study)	Prospective longitudinal studies to explore developmental pathways. Consider neurotoxic impairment	Reduce harms of substance abuse, foster abstinence, prevention for children of substance abusers
Which patterns are linked to SUD?	Different patterns, mainly very insecure	Conflicting (33 cross-sectional studies, biased by use of different measures)	Prospective longitudinal studies to explore different developmental pathways and brain correlates	Consider different patterns in the treatment of SUD
Do users of different substances differ?	Hints at differences, opioid users extremely insecure	insufficient	Systematic comparisons of users of different substances, consider substance related effects	Consider different substances in the treatment of SUD
Is severity of SUD linked to insecurity?	Inconclusive, no difference between studies in abuse vs. addiction samples, possible link in opioid abusers	Conflicting (36 studies, no systematic assessment of severity)	Systematic assessment and comparison of severity	Assess and consider severity in the treatment of SUD
What is the role of comorbid disorders?	Seem to be highly relevant, but data are inconclusive	Conflicting (seven studies)	Systematic assessment and comparison of comorbid disorders and brain correlates	Assess and consider comorbid disorders in the treatment of SUD
Are there differences in different age groups?	Closer relation in adolescence, inconclusive with regard to attachment patterns	Moderate (meta-analytic data)	Focus on adolescence, developmental processes, use family systems framework	Early intervention in adolescence, family systems approach

### Implications for Treatment

Based on the results of this review, some implications for the treatment and prevention of SUD will be discussed. We still are at an early stage, lacking an integration of attachment in a model of SUD, lacking treatment concepts, and clinical trials.

Results suggest that treatment approaches should consider insecure attachment in SUD patients. Since there seem to be different types of insecure attachment, these should be assessed and become part of individual treatment planning in the same way as information about consumed substances, level of severity, and comorbidities is used. Attachment theory stresses the therapeutic alliance as a means to develop more attachment security. However, establishing such a relationship with insecure substance abusers is difficult. It will often require specific engagement strategies, and it needs to be adapted to the individual pattern of attachment. Fowler et al. (82) found higher rates of treatment retention in addicts with preoccupied patterns. It seems to be more difficult to establish a therapeutic relationship with avoidant or unresolved individuals. Data show that substance-abusing patients with BPD are more avoidant and more difficult to reach for treatment (64).

Abstinence is a precondition for most treatments and for forming a therapeutic relationship. From an attachment point of view, abstinence means that substance abusers have to do without their usual coping strategy, leaving them without any functioning strategy. At the same time they are asked to open up to others, a subjectively dangerous step, considering negative relationship expectations. So therapists need to monitor their patients’ limited ability to get and stay in touch. From this perspective, relapses and treatment dropouts can be seen as avoidance of relationships.

Attachment-based approaches of individual treatment could be adopted for the treatment of SUD. To date, the most promising approach is mentalization-based therapy (MBT) (6). MBT is fostering the ability to mentalize, that is, to explore inner states of oneself and others. Preconditions of this ability are abstinence and felt security. The problem is that substance abusers usually do not feel secure at all when they reach abstinence. MBT for SUDs then has to take careful small steps, fostering security, keeping abstinence, and slowly exploring feelings and inner worlds. An ongoing RCT is currently evaluating MBT in a sample of opioid dependent adults in Sweden (17).

Longitudinal data show a bidirectional relation between insecure attachment and SUDs. This might have implications for treatment as well as prevention. It might become a vicious circle worsening both problems and a very challenging task to break this circle. Treatment has to focus on two goals that might reinforce each other in a negative or in a positive way. Quitting substance abuse will be easier when attachment security is fostered. The development of security, on the other hand, will benefit from abstinence. Gidhagen et al. (62) showed that it is possible to approach both goals successfully. They found an increase in attachment security in the course of addiction treatment.

The treatment of SUDs might help to prevent the development of even more insecure attachment. This should have a positive effect on relationships of substance abusers, including caregiving relationships with their children. Attachment-based prevention programs for children of substance-abusing parents are among the most elaborated and best evaluated approaches in the field (20). With regard to the prevention of SUDs, results suggest that fostering attachment security in childhood and adolescence might be effective. The importance of adolescence in the development of both attachment and SUD calls for early interventions designed for this age group. Among other things, this will need a family systems framework [Lewis (in this *Frontiers Research Topic)*]. Family treatments give a chance to treat attachment-related disorders in the context in which they have developed. Family therapy approaches for adolescent substance abusers are among the best evaluated treatments (83, 84). To date, there are two explicitly attachment-based approaches, attachment-based family therapy (85) and mentalization-based family therapy (MBFT) (86). Although neither of these focuses on SUDs, it seems possible to integrate attachment-focused work into family therapy approaches for SUDs (87).

Finally, attachment research has stimulated the search for new medications, pointing toward the importance of oxytocin. This substance is now considered a promising therapeutic agent for alcohol use disorders (88).

### Strengths and Limitations

This review has tried to give a concise overview over 30 years of research in the field. Since 2005, the number of studies has tripled, providing strong evidence for the general link between attachment and SUD. Meta-analytic and longitudinal evidence shows the interaction between attachment and SUD. Although results are still inconclusive in many regards, they indicate the need to differentiate between different patterns of attachment, different substances, comorbidities, and age groups. Results show the potential relevance of attachment within a multifactorial model of SUDs. But there will still be a lot of theoretical and empirical work to be done to integrate it into a concise model. Methodological problems in the assessment of attachment and substance abuse limit comparability. There is a tendency in many studies to focus on attachment as a single variable and to disregard its context and possible confounds. Future research will have to compare different groups of substance abusers systematically, including severity of substance use and comorbid disorders, linking psychological and neurobiological measures. We will need more longitudinal studies covering longer periods of time to completely understand the developmental pathways from attachment to SUDs. This review has not considered family systems of substance abusers or preventive aspects for children of substance-abusing parents. We will have to move to the level of systems and integrate family contexts into the study of attachment, linking attachment representations with relationship behavior and substance abuse.

## Author Contributions

The author confirms being the sole contributor of this work and has approved it for publication.

## Conflict of Interest

The author declares that the research was conducted in the absence of any commercial or financial relationships that could be construed as a potential conflict of interest.
